# Reproducibility and Validity of A-Mode Ultrasound for Body Composition Measurement and Classification in Overweight and Obese Men and Women

**DOI:** 10.1371/journal.pone.0091750

**Published:** 2014-03-11

**Authors:** Abbie E. Smith-Ryan, Sarah N. Fultz, Malia N. Melvin, Hailee L. Wingfield, Mary N. Woessner

**Affiliations:** Applied Physiology Laboratory, Department of Exercise and Sport Science, University of North Carolina, Chapel Hill, North Carolina, United States of America; Scientific Directorate, Bambino Hospital, Italy

## Abstract

Identifying portable methods to measure body composition may be more advantageous than using body mass index (BMI) to categorize associated health consequences. Purpose: To compare the validity and reliability of a portable A-mode ultrasound (US) to a criterion three compartment model (3C) for the measurement of body composition. Methods: Forty-seven overweight and obese subjects participated in this study. Body composition was measured once via air displacement plethysmography for body density (Bd) and bioelectrical impedance spectroscopy for total body water (TBW) for the 3C calculations. Ultrasound measurements (BodyMetrix, Intelametrix) were made using an A mode, 2.5- MHz transmitter. All measurements were made on the right side of the body at 7 skinfold sites. The US software calculated percent body fat (%BF), fat mass (FM) and fat free mass (FFM) from the 7-site Jackson and Pollock equation. Results: %BF and FM, respectively, measured by the US (29.1±6.5%; 27.4±8.1 kg) was significantly lower compared to the 3C model (33.7±7.6%; 31.8±9.8 kg; p<0.0005). Fat free mass was significantly higher for the US (66.7±13.0 kg) compared to the 3C model (62.3±12.6; p = 0.001). The US demonstrated respectable reliability for %BF, FM, and FFM with intraclass correlation coefficients (ICC) ranging from 0.84–0.98 and standard error of the measurement (SEM) values and 2.2%BF, 1.9 kg, 1.9 kg, respectively. Discussion: The US was found to under predict %BF and FM with large deviations from the criterion (n = 10>4%BF error). While the US was not valid in this population, it was reliable producing results with minimal error, suggesting this technique may be effective for tracking changes in a weight loss or clinical setting.

## Introduction

Obesity-related health complications have received increased federal attention due to the rising occurrence and associated medical costs. The National Health and Nutrition Examination Survey (NHANES) indicates that approximately 68% of US adults are overweight or obese [1], with 35.7% of those being classified as obese [2]. Trends in obesity continue to climb; it is predicted that by the year 2030, there will be approximately 65 million more obese adults in the U.S. [3]. Additionally, combined medical costs associated with obesity-related diseases such as diabetes, heart disease, stroke and cancer will increase by $48–66 billion per year in the U.S. [3]. The obesity epidemic has given rise to the need for accurate field-based measures of body composition at an individual level in order to better assess a patient’s health risks. An appropriate classification of body composition, specifically fat distribution, may allow for an improved evaluation of an individual’s overall health status [4], [5]. Additionally, clinical settings, such as doctors’ offices and weight loss facilities, may benefit from utilizing accurate field based measurements of percent body fat (%BF) in order to track weight changes over time, and to more effectively identify health risks.

Multi-compartment body composition measurement models have gained increasing support as criterion methods [6]. Specifically, the Siri three compartment (3C) model is considered a criterion due to its ability to account for variation in subject hydration by adding total body water (TBW) [7]. A widely used 3C model, incorporating proposed by air displacement plethysmography via the BodPod to predict body density (Bd), and bioelectrical impedance spectroscopy (BIS) to predict TBW has been shown to be accurate in predicting %BF in an overweight and obese population [8], a healthy population [9] and an athletic population [10].

A-mode, or amplitude mode, ultrasound (US) technology has been reported to produce accurate measures of %BF in normal weight subjects [11], [12] and an athletic population [13]. Furthermore, A-mode US has been previously validated against dual energy x-ray absorptiometry (DEXA) for determining %BF in healthy individuals [12], [13], and has been discussed as a feasible clinical tool due to ease of use [14]. US technology has been used for measurement of tissue thickness for decades [15], [16], [17]; however, the technology is not widely utilized for body composition, and more recent equipment employs a variety of beam frequencies. A-mode technology utilizes a narrow beam to scan tissue borders, represented by a change in amplitude of the signal. B-mode, or brightness mode, technology has been more commonly utilized, providing a two-dimensional image with greater resolution. To our knowledge, there is only one US device that was designed to measure body composition, using A-mode technology. This device is a portable, inexpensive field-based device, equipped with body composition software to measure fat mass (FM), fat free mass (FFM), and %BF. Currently, only two studies have demonstrated the accuracy of this device for estimates of %BF [11] and FFM [18] in young, healthy populations. There is no available data validating this device against a criterion method for body composition variables (%BF, FM, FFM), nor has it been evaluated in an overweight or obese population. Therefore, the purpose of this study was to assess the validity and reliability of A-mode ultrasound for the measurement of body composition in overweight and obese patients.

## Materials and Methods

### Subjects

Forty-seven subjects (20 male, 27 female; mean ± SD; Age: 37.6±11.6 years; Body Mass: 94.1±16.1 kg; Height: 172.9±10.1 cm, BMI: 31.5±5.2 kg⋅m^2^; Table 1), volunteered to participate in this study, approved by the University of North Carolina Chapel Hill Biomedical Institutional Review Board (IRB). All methods were conducted in accordance with the Declaration of Helsinki; participants signed an approved written informed consent, in compliance with IRB procedures. Subjects were not eligible for the study if they had any ongoing/untreated disease such as cancer or coronary heart disease, on medication known to affect hydration status; if they were pregnant or lactating; or if they had a history of weight loss surgery. All subjects used in the statistical analysis met the inclusion criteria: subjects were between the ages of 18 and 55 years and had a body mass index (BMI) between 25 and 50 kg⋅m^2^.

**Table 1:** Descriptive Statistics of all subjects, male, female, overweight and obese subjects, classified by BMI (Mean ±SD).

	Total (n = 47)	Male (n = 20)	Female (n = 27)	Overweight (n = 27)	Obese (n = 20)
Age (yrs)	37.6±11.6	40.8±10.8	35.2±11.8	38.8±11.3	35.9±12.0
Height (cm)	172.9±10.1	181.9±7.5	166.3±5.6	173.9±10.8	171.7±9.3
Weight (kg)	94.1±16.1	101. 3±13.3	88.8±16.1	85.1±11.2	106.3±8.1
Body Fat (%)	33.7±7.6	35.0±8.1	32.8±7.2	31.3±6.2	36.9±8.1
BMI (kg·m^2^)	31.5±5.2	30.6±4.4	32.2±5.7	28.1±1.3	36.2±4.6

### Protocol

All body composition measurements were taken on two separate days within 24 to 72 hours of each other. Measurements were not performed in a specific order, but were performed by the same trained investigator, at the same time (±2 hrs) of the morning. Subjects were asked to follow the same pre-testing guidelines for both sessions; which included an eight hour fast, water intake was allowed one hour prior to arrival, and abstention from exercise 12 hours prior to testing. Subjects were measured for height using a portable stadiometer (Perspective Enterprises, Portage MI, USA), and weight measured using a mechanical scale (Detecto, Webb City, MO, USA). Percent body fat, FM, and FFM was measured using an A-mode ultrasound (US; Body Metrix, Intelametrix, Livermore, CA, USA) and Siri 3C criterion as described below.

### Ultrasound (US)

**Figure 1:**
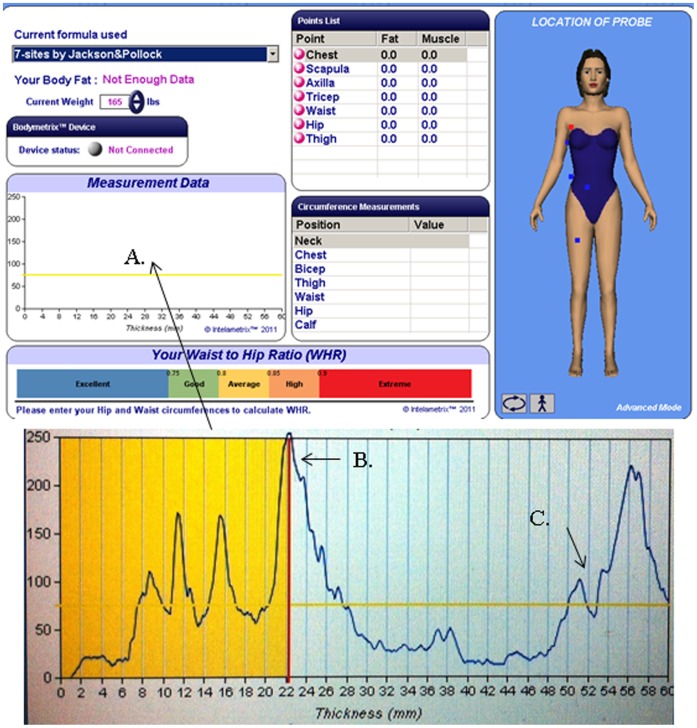
Image of the tissue boundaries and corresponding amplitudes produced from the proprietary software. Amplitudes appear within measurement data section (A). With a minimum of two measurements averaged. The fat-muscle boundary is illustrated at the first peak (B). Artifact within the muscle is demonstrated by other peaks (C).

Ultrasound measurements were conducted using the BodyMetrix BX-2000 A-mode ultrasound (US; BodyMetrix, Intelametrix, Livermore, CA), with a standard 2.5- MHz probe to measure subcutaneous fat thickness [18]; the higher the frequency of the probe, the greater the resolution. The principle of A-mode US utilizes a pulse-echo technique in which a pulse is applied at a speed of sound in the tissue [19], [20]. A-mode devices use a single beam, in a single plane, to determine the acoustic reflection and impedance of different tissue borders. Higher signal amplitudes/sound reflections result at tissue boundaries: skin-subcutaneous fat border, fat-muscle tissue boundary, as well as the muscle-bone tissue boundary (Figure 1). The change in amplitude is a result of the speed of sound, impedance of the measured tissue due to density, and attenuation of the beam. The proprietary software of the device used in the current study assumes an acoustic reflection of 0.012 for the fat-muscle tissue boundary and 0.22 for the muscle-bone boundary [19]. Sources of thickness error, particularly for fat, include errors in sound speed (±3.5%; <1% for same site and person); compression of fat (∼3%); and errors in electronics (<0.2%). Furthermore, a fatty muscle could potentially result in higher error.

**Figure 2:**
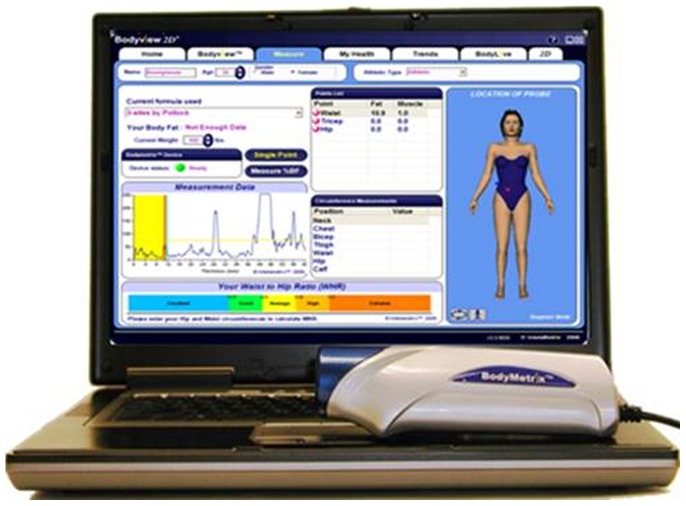
Image of the ultrasound transducer and corresponding software used for evaluation.

The probe was connected by USB to a standard laptop with corresponding proprietary software (BodyView Professional Software). Measurements were taken on the right side of the body while the subject was standing using seven-site skinfold locations according to Jackson and Pollock [21], and as instructed by the image on the computer screen. The measurement sites included: triceps, subscapular, abdomen, suprailiac, midaxillary, chest, and thigh. Measurements were made by applying transmission gel to the probe and lightly placing the probe perpendicular to the site. Measurements were taken at each site with minimal movement of the probe across the skin (+/−5 mm), and care was taken to control the pressure of the probe on the skin to ensure minimal tissue deformation. The subcutaneous fat thickness was calculated by the device software, using a linear relationship between US propagation velocity and the time of flight [12], [22] (Figure 2). Each site was measured approximately two to three times, based upon the software’s agreement between measurements, and the average of these trials was used to represent the final thickness measurement. These site specific subcutaneous fat thickness values were used to calculate %BF using the Jackson Pollock 7-site skinfold equation [21]. Fat mass and FFM were calculated from body mass and fat mass values using the following equations: %BF×body mass (BM; kg) = FM; and BM–FM = FFM.

### Siri 3-Compartment Criterion Measurements

#### Air displacement plethysmography

Body density (Bd) was determined using the BodPod® (Life Measurements Inc. Concord, California, USA), which measures body volume based on the inverse relationship between air volume and pressure under isothermal conditions [23], represented via Boyle’s Law (P_1_/P_2_ = V_2_/V_1_). Prior to testing, the BodPod was calibrated using a two-point calibration according to manufacturer’s instructions. It was first calibrated with the chamber empty, and then with a known 50L volume cylinder. Prior to testing, participants were asked to remove all metal including jewelry, watches, and glasses. Subjects also wore a swim suit or tight fitting spandex and a swim cap. Body mass was measured to the nearest 0.01 kg using the system’s calibrated electronic scale (Tanita Inc., Arlington Heights, IL, USA). Subjects were then instructed to sit quietly in the chamber in an upright position, to breathe normally and to keep their hands folded on their lap and feet planted on the floor. A minimum of two trials were performed and if the measurements were not within 150 ml of each other a third trial was conducted. The thoracic gas volume of the subjects was predicted using the manufacturer’s software based off standard prediction equations. Previous reports have shown that predicted lung volumes are not significantly different than measured volumes, even in obese subjects [24], [25].

#### Bioelectrical impedance spectroscopy

Bioelectrical impedance spectroscopy (SFB7 ImpediMed, Queensland, Australia) was used to estimate total body water (TBW) following the manufacturer’s recommendations. The BIS measures TBW by sending a current through the body and measuring the resistance or impedance (Ω) to that current [26]. The BIS has been shown to produce valid estimates of TBW when compared to criterion methods using deuterium oxide [27], [28]. Total body water measurements were taken while the subject was lying in a supine position on a non-conductive surface, with arms and legs not touching following a five minute rest period. Before placing the electrodes on the skin, all excess hair was shaved and the area was cleaned with alcohol and gauze, to remove any interference. Two electrodes were placed 5 cm apart on the right hand and wrist. The first electrode was placed superior to the wrist, medial to ulnar head; the second electrode was placed 5 cm from that electrode, proximal to the third metacarpophalangeal joint [29]. Two electrodes were placed 5 cm apart on the right foot and ankle, the first electrode was placed superior to the ankle between the lateral and medial malleoli; the second electrode was placed 5 cm away, proximal to the second metatarsophalangeal joint. Measurements were repeated twice, and the average was used to determine each participants TBW value. The Bd and TBW values were then used to calculate %BF using the Siri, 3C model equation [7]: %BF = [(2.118/Bd–(0.78×TBW/BM (kg))–1.354]×100.

### Statistical Analysis

All demographic data are presented as mean ± SD values and listed in Table 1. A paired samples t-test was performed in order to determine if there was a significant difference between US measurements of %BF, FM and FFM and the criterion 3C model. Bland and Altman plotting was performed for the validity assessment. The difference between each body composition variable (%BF, FM, LM) was plotted against the mean value from the US and 3C model, for each respective variable. Normal, overweight, and obese classifications were established based on BMI cut points previously described by Gallagher et al. [30]. Similar classifications were established from US %BF and 3C%BF (criterion) [31] to determine the utility of US compared to BMI for health identification.

Test-retest reliability was examined using model 2,1 [32] to determine intraclass correlation coefficients (ICC), the standard error of the measurement (SEM), and the minimum difference (MD) score using a custom written Excel (Microsoft Inc., Redmond, WA) spreadsheet. The ICC was calculated with the following equation [33]:
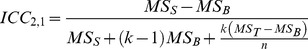
(1)


*MS_S_* represents the mean square for subjects, *MS_E_* is the mean square error, *MS_T_* is the mean square for trial, *k* represents the number of trials, and *n* is the sample size. The SEM for this model was calculated using the following equation [34]:
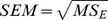
(2)


The MD score, also referred to as the minimal detectable change, may be important for clinicians evaluating whether a difference or change (as a result of an intervention or treatment) in body composition can be considered ‘real,’ as described in the review by Weir et al. [33]. The MD was calculated using the following equation:
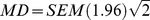
(3)


All statistical analyses were performed using SPSS 20.0 Statistical Analysis Software (IBM, Somers, NY, USA).

## Results

### Validity

**Figure 3:**
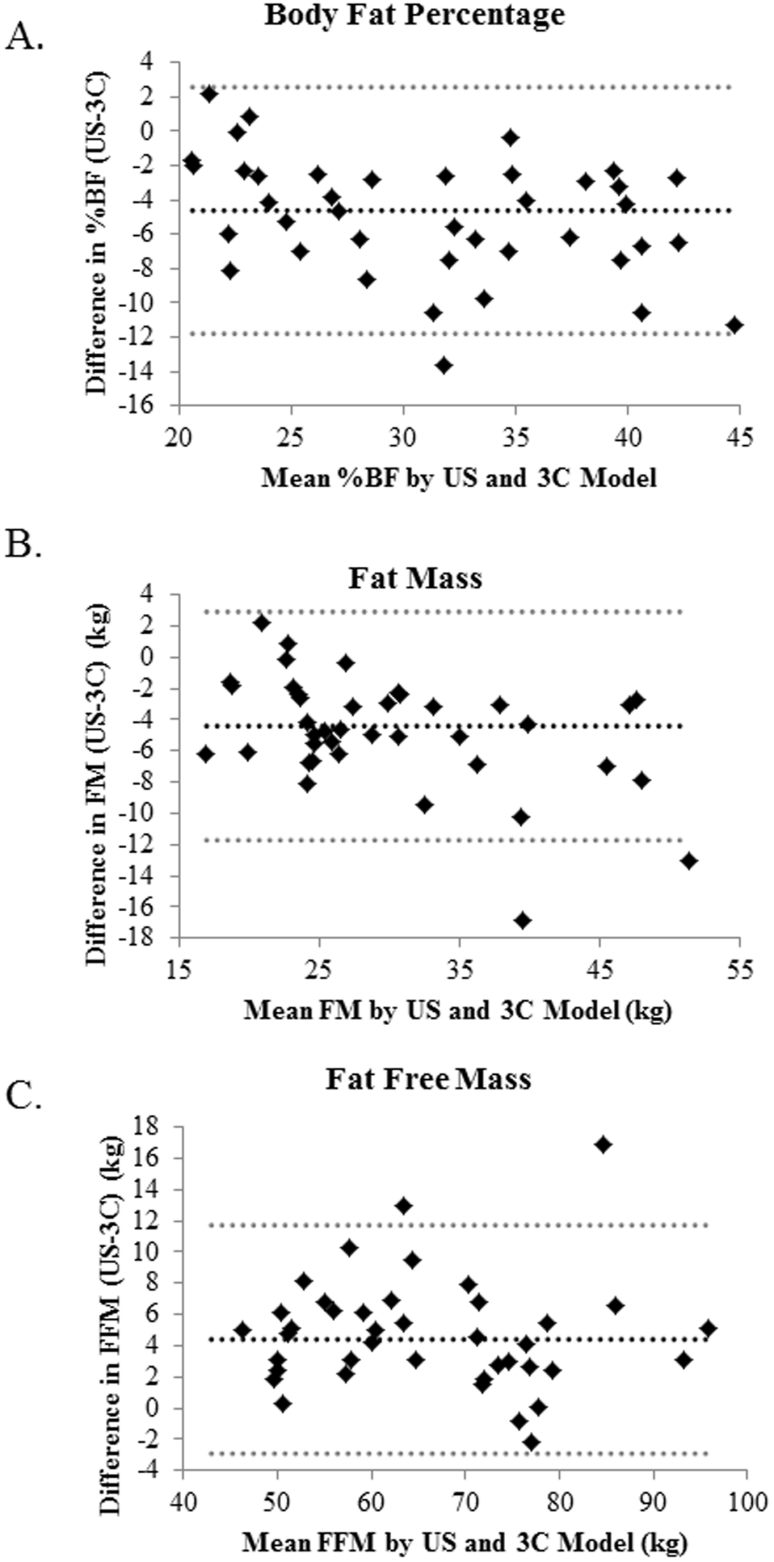
Bland and Altman plots comparing individual differences in %BF (A), FM (B), and FFM (C) measured from the ultrasound (US) - 3compartment model (3C) methods compared with the mean values for both methods.

All body composition variables measured from the US were significantly different compared to the 3C criterion model; %BF (P = 0.001); FM (P = 0.001); FFM (P = 0.001) (Table 2). A significant difference was also seen between the US and 3C model for all variables when stratified by overweight and obese categories (Table 3). The agreement between US and 3C models for %BF, FM, and FFM are depicted in Figure 1A–C. While there appears to be a significant difference between US and 3C values, the majority of the values were within the limits of agreements between the two methods and there was no significant systematic bias (Figure 3). Classification differences between normal, overweight and obese identifiers determined from BMI, US%BF, compared to the3C%BF criterion, revealed that the US misclassified 29.8% of the total subjects, in comparison to 72.3% from BMI (Table 4).

**Table 2:** Comparison of percent body fat (%BF), fat mass (FM) and fat free mass (FFM) between the ultrasound (US) and 3- compartment model (3C). (Mean±SD).

	%BF		FM		FFM	
Method	X ± SD	P Value	X ± SD	P Value	X ± SD	P Value
US	29.0±6.5	0.001	27.3±8.1	0.001	66.7±13.0	0.001
3C	33.7±7.6		31.7±9.8		62.3±12.6	

**Table 3:** Comparison of percent body fat (%BF), fat mass (FM) and fat free mass (FFM) between the ultrasound (US) and 3- compartment model (3C) for overweight and obese subjects. (Mean±SD).

			%BF		FM		FFM	
	n	Method	X ± SD	P Value	X ± SD	P Value	X ± SD	P Value
Overweight	27	US	27.1±5.7	0.001	22.4±3.9	0.001	62.3±11.7	0.001
		3C	31.3±6.2		26.2±4.5		58.8±11.9	
Obese	20	US	31.7±6.8	0.001	33.6±8.1	0.001	72.6±12.9	0.001
		3C	36.9±8.3		39.2±10.2		67.0±12.5	

**Table 4:** Classification of ‘normal weight’ versus ‘overweight’ versus ‘obese’ for BMI and US, compared to the 3C %BF criterion.

Inclusion BMI Classification	BMI Classification	US%BF Classification	3C%BF Classification
Obese (BMI = 30.0–46.0)	20	14	22
Overweight (BMI = 25.0–29.9)	27	13	11
Normal weight (BMI = 18.5–24.9)*	0	20	14
Correctly classified	13/47 (27.7%)	33/47 (70.2%)	

*Normal BMI was an exclusion criteria.

### Reliability

Ultrasound %BF reliability from day 1 (mean ± SD; 28.9±6.8%) and day 2 (29.3±6.3%) were not significantly different (P = 0.284). The relative consistency (ICC_2,1_) for %BF was 0.98; standard error of the measurement (SEM) was 2.2%BF, and MD was 4.3%BF. Between days, FM and FFM values were trial 1: FM = 29.6±7.7 kg; FFM = 61.2±13.0 kg, and trial 2: FM = 26.0±7.2 kg; FFM = 65.0±13.0 kg. The relative consistency values (ICC) for FM and FFM were 0.93 and 0.98. Consistency values SEM were 1.8 kg (FM) and 1.9 kg (FFM). Minimal Differences values for FM and FFM were 5.2 kg and 5.3 kg, respectively.

## Discussion

To the best of our knowledge, this is the first study to investigate the validity and reliability of the A-mode US (US; Body Metrix, Intelametrix, Livermore, CA) in an overweight and obese population. The primary finding of this investigation demonstrated that there was not an agreement between the US and the 3C model, indicating that the US significantly under-estimated %BF in an overweight and obese population. Due to the variability in body fat measurements, this device may not be the best body composition for a single point assessment. However, previous papers suggest that there is a strong agreement between the A-mode device and skinfolds [20], [35], potentially supporting the use as a portable field-based method. Additional results demonstrated acceptable reproducibility for US when measuring %BF, FM, and FFM, reporting an average 2% error in percent body fat between day measurements. The reliability of the US device did not seem to be influenced by upper-end variations in fat (overweight vs. obese; Table 2). Due to the high reliability and portability of the US, it may be an effective clinical tool for baseline classification of health, in comparison to BMI. The US misclassified 30% of the total subjects in comparison to the 3C model, while 72% of subjects were misclassified according to BMI.

Few studies have identified valid and reliable methods for body composition measurement in an overweight and obese population. Ginde et al. [36] found that BodPod was a valid method of measuring body density compared to under water weighing (UWW) in an overfat population (overweight: Δ0.004±0.007, obese: Δ−0.001±0.007, severely obese: Δ0.001±0.007). Likewise, the BIS has been shown to be valid in tracking changes in body composition, and is useful for group comparisons, but has not been shown to be valid in measuring body composition in the overweight and obese population [29], [37], [38], [39], [40]. While these devices have been shown to be accurate measures for estimating %BF in various populations, additional portable field-based methods, such as the US could be clinically advantageous. The current study is the first to report data using the BodyMetrix US for measuring %BF, FM or FFM in an overweight and obese population. This device under-predicted values for %BF (Δ4.7±1.1%) and FM (Δ10.4±1.7 kg), while over-predicting FFM (Δ4.4±0.4 kg) when compared to the 3C criterion. While a 4.7%BF significance (p<0.05) between US and 3C criterion should not be discounted, all body composition measurements result in error. Specifically, previous comparisons in overweight subjects have reported a 5.3%BF difference between a 4 compartment criterion and DEXA [41]. Similar significant differences were also reported for comparisons of BodPod, DEXA and BIA to a 3C criterion in obese subjects [42]. Therefore, the US may have some practical clinical use. However, it should be noted in the current study, 10 subjects values resulted in an under-prediction of %BF by greater than 4%, in comparison to the 3C criterion; while 16 subjects were within a 4%BF agreement with the criterion. Therefore it is likely (21%) that subjects may result in a %BF value that is more than 4% different than other more sophisticated methods.

In contrast to the present study, previous studies have demonstrated validity of the US, but strictly with healthy [11], [12], [13], [43] and athletic [13], [18] populations, as well as with varying US technologies (A- mode, B-mode, M-mode). Johnson et al. [11] reported that the US (BodyMetrix) was valid compared to BodPod (Δ0.2±0.69%) and BIS (Δ0.4±3.29%BF) in healthy, college age individuals. Likewise, Pineau et al. [12], using an A-mode US (Lecoeur Electronique Co.,Chuelles, France), found the device to produce accurate measures of %BF in relation to the DEXA. Utter et al. [18] is the only previous study to use the BodyMetrix US to measure FFM, supporting its validity compared to hydrostatic weighing (Δ0.2±0.1 kg). However, the subjects measured were high school wrestlers, which could influence the generalizability to the population in the current study. More so, physiological differences in overweight and obese populations must be considered when US validation data are compared. A greater amount of adipose tissue and greater inconsistencies within the tissue of an overweight and obese population could cause a slower pulse through the subcutaneous adipose tissue, initiating an uneven reflection of the pulse to return back to the probe [12], [22], [44]. This in turn could skew the image and measurement of tissue depth made by the US, and increase error in an overweight and obese population. Furthermore, fatty muscle could create error when determining the fat-muscle tissue border. This was likely the case in the current study with an under-prediction of FM and over-prediction of FFM. Additional research is warranted to explore its validity in a larger sample size. Based on the preliminary data presented here, there were no differences in validation for overweight vs. obese, potentially suggesting that varying degrees of excess fat, beyond a certain point (>25 BMI) does not make the US less accurate.

The current study is the first to report the sensitivity of the BodyMetrix US for measuring %BF in an overweight and obese population. Results are similar to those of Stolk et al. [45] who found US (ATL HDI 3000, M-mode, System, Bothell, Washington, USA) to be valid and reliable in measuring intra-abdominal adipose tissue in a group of 19 obese subjects compared to computed tomography (CT) scan (p<0.001, Δ = 0.4±0.9cm, %CV = 5.4%). Data from the current study demonstrate acceptable reproducibility of measurements using the BodyMetrix US with a high ICC (0.98) and low standard error of measurement (2.2%BF), indicating that the US could be used to track body composition changes in overweight and obese individuals. Reproducibility for FM and FFM were also high, suggesting the US may also be sensitive enough to detect these compartment changes. Due to the high reliability, this A-mode US may be useful for field-based evaluations, such as during a weight loss program or clinical testing where body composition will be measured multiple times. This device may also be useful in epidemiological data collection, due to its ease of use and low cost (∼$2,000).

Although results show that the BodyMetrix US will likely under-predict %BF beyond a reasonable amount (>4%), it may be useful for multiple measurements and tracking changes over time, thereby giving an accurate picture as to the change in %BF, FM, and FFM for a given individual. In the current study, while the same investigator performed all measurements for each subject, technician variability may influence measurements due variability in force applied. Specifically, limitations of this particular device are described by Wagner [14]. Furthermore, as a result of the novelty of this US, there is minimal data to compare the US with other body composition devices, as well as a lack of standardization for measurement procedures (i.e. probe pressure); inherent artifacts, such as intramuscular fat and fascia may influence the accuracy of the probe to accurately detect correct tissue-border interfaces. Due to a large amount of adipose tissue and tissue inconsistencies in an overweight and obese population, the US could under-predict %BF; more studies are needed to investigate the validity of the US in a larger sample size and broader spectrum of body composition (BMI 18.5–45.0 kg/m^2^). It would also be valuable to compare this field-based technique directly with other field-based methods (i.e. skinfolds, BIA, etc). Another limitation of the current study lies within the use of predicted lung volume for BodPod estimates, which may account for some variability in method comparisons.

## Conclusion

The comparison of %BF using the US against the 3C model demonstrated that the US significantly under-predicted %BF and FM, and over-predicted FFM in an overweight and obese population, regardless of BMI; however, it was reliable across varying measurement time points. Due to the reproducibility reported, the US may be useful in a clinical setting for tracking changes in body composition over time. The reported SEM was lower than laboratory methods (underwater weighing, DEXA, BIS), making it a practical portable clinic method. The US misclassified approximately 30% of the subjects, in comparison to a 3C %BF classification for overweight/obese categories, but it may be more effective for classification than BMI (Table 4). Additionally, there was a 21% (n = 10) under-prediction of %BF beyond an acceptable error (>4), therefore if used as a one-time assessment, results should be interpreted with caution. Due to the advantages of the US: affordability, portability and ease of use, it may be beneficial to use in a clinical setting, such as physician clinics, weight loss facilities or gyms, in order to obtain a better assessment of body composition than BMI or skinfolds. Future research should evaluate the use of this portable A-mode US in a larger sample to improve the generalizability of these results, as well as extend the comparisons to other field-based techniques.
